# The reconstruction of invasion histories with genomic data in light of differing levels of anthropogenic transport

**DOI:** 10.1098/rstb.2021.0023

**Published:** 2022-03-14

**Authors:** J. Hudson, S. D. Bourne, H. Seebens, M. A. Chapman, M. Rius

**Affiliations:** ^1^ School of Ocean and Earth Science, University of Southampton, National Oceanography Centre, European Way, Southampton SO14 3ZH, UK; ^2^ Senckenberg Biodiversity and Climate Research Centre, Senckenberganlage 25, 60325 Frankfurt, Germany; ^3^ Department of Biological Sciences, University of Southampton, Life Sciences Building 85, Highfield Campus, Southampton SO17 1BJ, UK; ^4^ Department of Zoology, Centre for Ecological Genomics and Wildlife Conservation, University of Johannesburg, Auckland Park 2006, South Africa; ^5^ Centre for Advanced Studies of Blanes (CEAB, CSIC), Accés a la Cala Sant Francesc 14, Blanes 17300, Spain

**Keywords:** biological invasions, genetic diversity, invasion routes, non-indigenous species, population connectivity, population genomics

## Abstract

Unravelling the history of range shifts is key for understanding past, current and future species distributions. Anthropogenic transport of species alters natural dispersal patterns and directly affects population connectivity. Studies have suggested that high levels of anthropogenic transport homogenize patterns of genetic differentiation and blur colonization pathways. However, empirical evidence of these effects remains elusive. We compared two range-shifting species (*Microcosmus squamiger* and *Ciona robusta*) to examine how anthropogenic transport affects our ability to reconstruct colonization pathways using genomic data. We first investigated shipping networks from the 18th century onwards, cross-referencing these with regions where the species have records to infer how each species has potentially been affected by different levels of anthropogenic transport. We then genotyped thousands of single-nucleotide polymorphisms from 280 *M. squamiger* and 190 *C. robusta* individuals collected across their extensive species' ranges and reconstructed colonization pathways. Differing levels of anthropogenic transport did not preclude the elucidation of population structure, though specific inferences of colonization pathways were difficult to discern in some of the considered scenario sets. We conclude that genomic data in combination with information of underlying introduction drivers provide key insights into the historic spread of range-shifting species.

This article is part of the theme issue ‘Species’ ranges in the face of changing environments (part I)’.

## 1. Introduction

The ever-increasing rate of globalization of trade is intensifying the anthropogenic transport of species [1,2], leading to introductions of many species to regions away from their native ranges. As non-indigenous species (NIS) cause major impacts on ecological communities around the world, understanding the underlying mechanisms facilitating NIS' spread is fundamental for biodiversity conservation and management [3]. One way of studying NIS’ spread is through identifying genetic patterns across different spatial scales [4–6]. Such studies have suggested that anthropogenic transport geographically reshuffles genotypes [7–10], and/or causes regional or global genetic homogenization [11–14]. Because unravelling colonization pathways is key for understanding NIS' spread [15] and for planning mitigation strategies [16], understanding how anthropogenic transport of species may dampen our ability to reconstruct invasion routes is fundamental.

Anthropogenic transport of species, by definition, increases population connectivity across species' ranges. The genetic composition of colonizing populations can be affected by numerous different processes and scenarios. For example, genetic bottlenecks and founder effects in recent colonizations may lead to population structure across the species range [17]. Conversely, genetic homogenization among populations may be expected if local adaptation within introduced ranges is weak, or if high levels of gene flow (through frequent introductions) persists [18]. Furthermore, the timing and magnitude of anthropogenic transport may affect population structure. For example, ‘recolonizations’ of introduced genotypes back to the native range may result in reduced genetic structure throughout the species range. A similar pattern of homogenization could also occur due to variation in effective population size, *N*_e_. Previous work has found a positive correlation between *N*_e_ in the introduced range and time since invasion [19]. Large *N*_e_ would prevent genetic drift, slowing divergence between populations, even in the absence of ongoing gene flow via continuing introductions. Conversely, an ancient invader might be expected to develop strong population structure throughout its range if local adaptation of introduced populations has evolved and/or if reduced gene flow has led to genetic drift. Another mechanism enhancing population structure may be through multiple introductions of genotypes from genetically divergent source populations, increasing the propensity for intraspecific genetic admixture [20]. Changes in transportation routes of species, in the absence of natural population connectivity, can also lead to a subset of introduced populations becoming disconnected from other populations, resulting in a rapid change in allele frequencies [21] or a reduction in genetic diversity due to drift [22].

High-throughput sequencing (HTS) enables scientists to obtain a substantial genomic coverage and capture patterns of genome-wide variation [23] and this HTS offers significantly higher resolution of fine-scale gene flow than studies analysing a few loci [24]. HTS has been used to reconstruct invasion histories [16,25,26], inferring the presence of multiple and sequential introductions [27,28], as well as revealing the presence of genetic admixture that may have fitness consequences on colonizing populations [25,29]. In addition, studies of neutral loci have analysed population genomic patterns of NIS in both introduced and native ranges [30,31], identified secondary contacts [32] and detected genetic bottlenecks [33]. However, no study using HTS has to date tested how anthropogenic transport of species affects our ability to infer colonization pathways of NIS [26].

Here we used a comparative approach to unravel the effects of different levels of anthropogenic transport on the reconstruction of introduction pathways using HTS data. For this, we studied two biologically similar sessile marine NIS that have widespread distributions but have presumably been affected by different levels of anthropogenic transport. Both species belong to the class Ascidiacea (phylum Chordata) and have limited natural dispersal capabilities with the duration of motile early life-history stages being only a few days [34,35]. Ascidiacea species are among the most prolific groups of invasive species on the planet [36], often causing negative economic impacts on important human activities [37]. We first analysed historical inter-regional shipping to detect patterns of anthropogenic transport among the regions where the study species were present. We then sequenced samples collected from across the ranges of the study species to explore range-wide connectivity patterns. Finally, we inferred the most likely colonization pathways using Bayesian methods and determined the putative impact of anthropogenic transport on our ability to reconstruct invasion routes.

## 2. Material and methods

### (a) Study species and field sample collection

We studied two ascidian species, *Microcosmus squamiger* (Michaelsen, 1927) and *Ciona robusta* (Hoshino & Tokioka, 1967) for which species records suggest differing levels of anthropogenic transport (electronic supplementary material, table S1). Briefly, *M. squamiger* is native to Australia [38,39] and was first reported outside of its native range in the mid-twentieth century in the Mediterranean Sea and South Africa [39,40]. *Ciona robusta* is putatively native to the northwest Pacific [41] and has been recorded in the Mediterranean Sea from the nineteenth century [42], followed by records in South Africa [43], northeast Pacific [44], Australia [45,46], New Zealand [47] and Hong Kong [48] throughout the twentieth century, and the south coast of England [49] since the early twenty-first century. Both species' population genetics have previously been studied using a relatively small number of genetic markers [31,41,42], and thus no study to date has reconstructed the invasion routes of these NIS using genome-wide tools.

**Figure 1.**
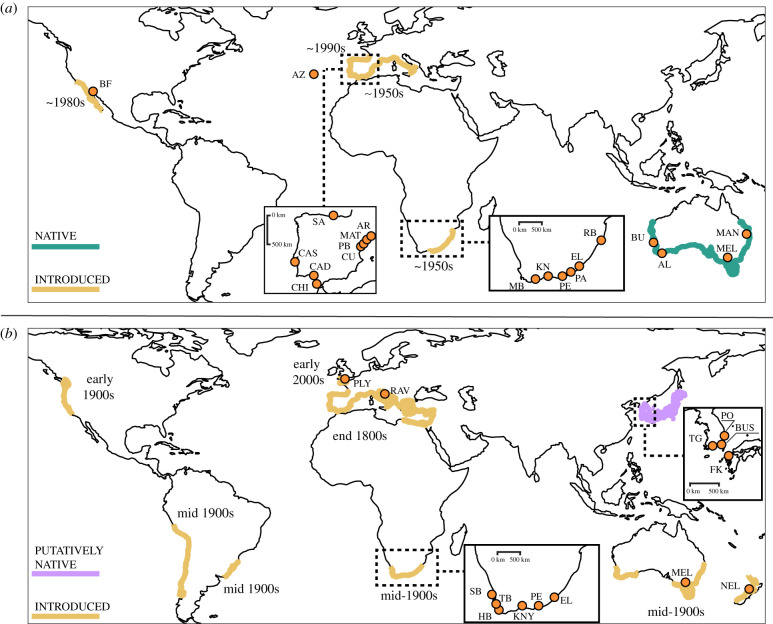
Sampling sites and ranges of (*a*) *Microcosmus squamiger* (boxes show enlarged Iberian and South African sites) and (*b*) *Ciona robusta* (boxes shows enlarged South African, Iberian and northwest Pacific sites). Coloured areas show status of their ranges and years next to each region when each species was first recorded as introduced. Orange dots indicate sampling sites (see electronic supplementary material, table S2 for full details of these sites). Site abbreviations are as follows: (*a*) BU, Bunbury; AL, Albany; MEL, Melbourne; BF, Bahía Falsa; AZ, Azores; SA, Santander; CA, Cascais; CAD, Cádiz; CHI, Chiclana; CU, Cubelles; PB, Port Barcelona; MAT, Mataró; AR, Arenys de Mar; MB, Mossel Bay; KNY, Knysna; PE, Port Elizabeth; PA, Port Alfred; EL, East London; RB, Richards Bay; (*b*) FK, Fukuoka; BUS, Busan; PO, Pohang; TG, Tongyeong; NEL, Nelson; MEL, Melbourne; KNY, Knysna; PE, Port Elizabeth; EL, East London; SB, Saldanha Bay; TB, Table Bay; HB, Hout Bay; RAV, Ravenna; PLY, Plymouth. (Online version in colour.)

We sampled individuals from both the native and introduced ranges of the study species (figure 1; electronic supplementary material, tables S3 and S4). Sampling sites were chosen to maximize distributional coverage and to include geographical areas that were not covered in previous genetic studies [31,42]. Specifically, we made a concentrated effort to sample regions where little sampling was conducted in previous studies (e.g. [42]), such as Australasia or South Africa (figure 1). At each site, we collected 20–30 individuals by hand from ropes and marina buoys/pontoons, or from artificial rocky substrata using SCUBA. We enforced a spacing of a few tens of centimetres among sampled individuals to minimize the collection of closely related individuals. We then dissected a piece of the mantle (muscle tissue) from each individual and immediately fixed the tissue samples in greater than 99% ethanol. Samples were then transported to the laboratory where they were stored at −80°C until DNA extraction.

### (b) Historical shipping data

We obtained historical shipping data from global regions across the study species' ranges. These data came from two independent datasets that spanned two sequential time periods: the Climatological Database for the World's Oceans (CLIWOC, 1750–1850, http://webs.ucm.es/info/cliwoc/) and the International Comprehensive Ocean-Atmosphere Data Set (ICOADS, 1865–2014, http://icoads.noaa.gov/). The CLIWOC dataset draws from digitized British, Dutch, French and Spanish ships' logbooks, with a focus on ships sailing in the Atlantic and the Western Indo-Pacific. The ICOADS data derive from various sources worldwide (http://icoads.noaa.gov/). Both datasets were originally constructed to reconstruct historical ocean and atmospheric conditions, and not shipping dynamics. As a result, they do not include all shipping activity, but give a good representation of general shipping dynamics at that time.

Both datasets provided ship location dates and geographical details during their travel, enabling the reconstruction of individual ship trajectories and shipping intensities. The CLIWOC dataset provided additional information about anchor points, which can be interpreted as port calls of that ship. The ICOADS dataset did not provide information about anchor points, and it was thus necessary to infer port calls from ship trajectories. To determine actual port calls, we calculated the shortest distance of each ship coordinate to a list of 1620 ports obtained from the World Port Index 26th Edition (https://opendata-esri-de.opendata.arcgis.com/datasets/esri-de-content::world-port-index/explore). We only considered large ports (i.e. not recreational marinas which are mostly recent developments) that we could assume persisted over the past 250 years. Geographical details of ship locations were only provided once a day and no records were available when a ship stayed in the actual port. We therefore considered a port call if a ship sailed within 10 km distance from a port. We checked individual ship trajectories and used different distances to test the sensitivity of the reconstruction of shipping routes. In total, we obtained 7238 individual ship movements from the CLIWOC dataset and 210 423 ship movements from ICOADS. For both datasets, the temporal and spatial coverage was not always consistent and thus data were only analysed on coarse temporal (50-year intervals) and spatial (regional) scales. To visualize historical shipping data, we created chord diagrams using the R package ‘circlize' [50], to show the number of direct ship travels between regions where the study species occur for each 50-year period between 1750 and 2000.

### (c) DNA extraction and genotyping

Total genomic DNA was extracted from all tissue samples using the ReliaPrep^TM^ gDNA Tissue Miniprep System (Promega, Madison, WI, USA). DNA was sent for sequencing at Cornell Genomics Diversity Facility (Cornell University, Ithaca, NY, USA). The restriction enzymes *Pst*I, *Eco*T221 and *Ape*KI were trialled to identify the one that created suitable libraries (fragments less than 500 bp, presence of non-repetitive DNA), and thus *Pst*I was used for *M. squamiger*, and *Eco*T221 for *C. robusta*. Genotyping was performed using the genotyping-by-sequencing protocol (GBS) [51], and took place on an Illumina HiSeq 2500, using single-end 100 bp reads.

### (d) Data processing

We processed data from each species independently using the same bioinformatics pipeline. Briefly, sequence data were first passed through FastQC [52] to investigate read quality. After successfully passing quality checks, the GBS reads were assembled de novo using ipyrad v. 0.7.30 [53] using parameters recommended for single-end GBS data (http://ipyrad.readthedocs.io/). We then conducted read assembly, single-nucleotide polymorphism (SNP) filtering and loci selection (see full description in the electronic supplementary material).

### (e) Genomic summary statistics, population structure and differentiation

Within-population indices of genetic diversity (expected heterozygosity (H_E_), observed heterozygosity (H_O_), and the inbreeding coefficient (F_IS_)) were calculated using the ‘diversity v.1.9.90' package [54] within R [55]. To provide a graphical representation of between-site genetic differentiation, and to test for population structure within our datasets, we used two genetic clustering methods. Firstly, we used the ‘adegenet v. 2.1.3' package [56] in R to perform a Principal Component Analysis (PCA) using the function *dudi.pca*. Secondly, we used the software ADMIXTURE v. 1.3 [57] to group individuals into one of *K* putative clusters, using a maximum-likelihood estimation. For both species, the number of tested clusters ranged from 1 to *n*, where *n* = the number of sites individuals were sampled from. The R package ‘hierfstat v. 0.5-7' [58] was used to calculate genomic differentiation, as inferred through pairwise-population values of *F*_ST_.

### (f) Combining genomic indices and shipping data

For each period of shipping data available we assessed the correlation between the number of shipping events (hereafter referred to as shipping intensity) and genomic differentiation. We grouped our study sites into regions corresponding to the spatial scale of our shipping data analysis, and calculated mean *F*_ST_ values of sites among these regions, before performing a Spearman's rank correlation between shipping intensity and *F*_ST_ in R using the package ‘ggpubr v. 0.4.0' [59].

### (g) Reconstructing colonization pathways

We used DIYABC Random Forest v. 1.0 [60], which uses Approximate Bayesian Computation to evaluate different evolutionary scenarios, to infer colonization pathways. For all scenarios, training sets were generated using 2000 simulations per model. Note that supervised machine learning methods such as random forest (RF) use all simulations to learn the mapping of data to models, and subsequently a smaller training set is required compared to ABC methods [60]. Current knowledge of the study species' global distribution and historical species records (figure 1) were used to inform model construction. In addition, the results of the PCAs and population differentiation were used to pool genomically similar geographical sites and guide the building of the models (for a detailed description of the model sets, see the electronic supplementary material). We identified the most likely scenario of each set using the ‘RF analysis' module of DIYABC-RF (see full details in the electronic supplementary material).

## 3. Results

### (a) Historical inter-regional shipping patterns

**Figure 2.**
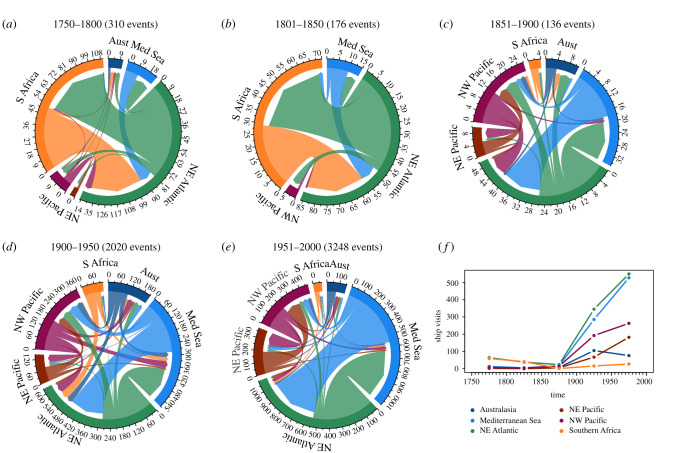
Temporal development of the global shipping network from 1750 to 2000, considering the regions where the study species can be found. (*a*–*e*) Chord diagrams showing the number of ship travel events between marine regions over approximately 50-year intervals. The arrows at the end of the flows represent incoming travel to that region. Each region is colour assigned and represented by a circular segment proportional to the respective shipping intensity. (*f*) Temporal development of the total number of ship visits at ports for each region. (Online version in colour.)

We found a clear pattern of increasing complexity and magnitude of global shipping over recent time (figure 2). Indeed, the total number of shipping events was small initially but showed a sharp increase from the beginning of the twentieth century, with the period between the years 1750 and 1800 containing 155 events, 1801–1850 containing 88 events, 1851–1900 containing 68 events, 1901–1950 containing 1010 events and 1951–2000 containing 1624 events. Among the regions of interest for this study, most intense shipping was consistently recorded in the northeast Atlantic, representing around 40% of shipping between 1750 and 2000 (figure 2*f*). South Africa was also a major shipping donor/recipient particularly before 1850 and was involved in minor shipping trade with the northwest Pacific prior to the 1800s. Shipping within the *M. squamiger* native range (i.e. Australia), while being present at low intensity in the eighteenth century, intensified from the mid nineteenth century onwards. Mediterranean shipping steadily increased from 1750, representing 20% of shipping traffic from the 1950s onwards. These shipping data indicate that from the 1750s, shipping was prevalent among regions across the range of *C. robusta* (Australia, Mediterranean Sea, northwest Pacific, northeast Pacific and South Africa; figure 2*b,c*). Thus, the combined used of historical shipping data and the species records (electronic supplementary material, table S1) suggested a longer history of anthropogenic transport in *C. robusta* compared to *M. squamiger*.

### (b) Genotyping of neutral single-nucleotide polymorphisms

We genotyped 365 *M. squamiger* and 214 *C. robusta* individuals from across their species ranges. Of these, 280 *M. squamiger* and 190 *C. robusta* successfully passed our sequencing QC (electronic supplementary material, tables S3 and S4). Following our filtering protocol, we retained 2115 SNPs and 3227 SNPs for *M. squamiger* and *C. robusta*, respectively. We then identified putatively non-neutral SNPs using BAYESCAN and pcadapt and removed those that were presented in either method, leaving a dataset of 1994 SNPs and 3139 SNPs for *M. squamiger* and *C. robusta*, respectively.

### (c) Genomic summary statistics

For *M. squamiger*, expected and observed heterozygosities were consistent across the range (native range mean H_E_ = 0.111 and mean H_O_ = 0.064; introduced range mean H_E_ = 0.114 and mean H_O_ = 0.065; electronic supplementary material, figure S1 and table S5) and the mean number of private alleles per site was greater in the native range (mean = 31.3 private alleles per site) than the introduced range (mean = 6.2 private alleles per site). For *C. robusta*, H_E_ was higher in the putative native ranges (mean H_E_ = 0.191) than in the introduced range (mean H_E_ = 0.148; electronic supplementary material, figure S2 and table S6); however, for Ravenna (the Mediterranean site) in the introduced range, H_E_ was higher than all other sites (0.241). The number of private alleles across the range showed the opposite pattern to H_E_, with sites within the native range having fewer private alleles (mean = 10.0) than the introduced range (mean = 35.6). All sites, for both species, exhibited positive F_IS_ values (for values of genetic diversity indices for each site, see electronic supplementary material, tables S5 and S6).

### (d) Population structure and differentiation

**Figure 3.**
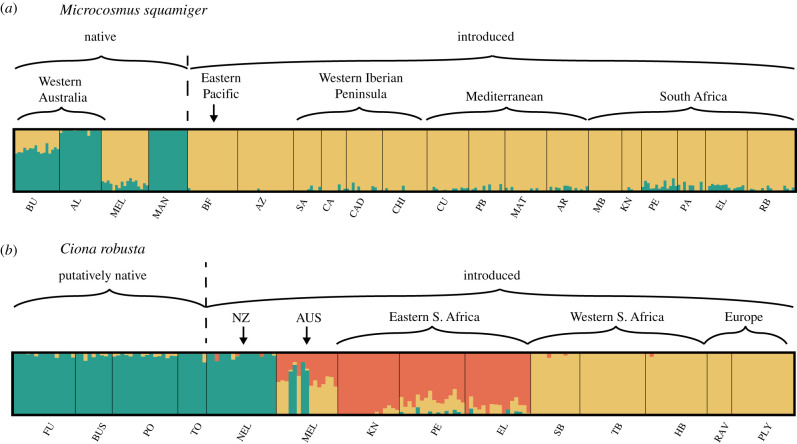
Genomic clusters within (*a*) *Microcosmus squamiger* (optimal number of clusters = 2) and (*b*) *Ciona robusta* (optimal number of clusters = 3) as inferred by ADMIXTURE v.1.3. Population abbreviations are given in figure 1. (Online version in colour.)

Genomic differentiation was high among native sites of *M. squamiger*, but low within the introduced range (figure 3*a*). The optimum number of clusters identified by ADMIXTURE was *K* = 2, with one cluster containing three native sites (BU, AL and MAN) and the second cluster containing the native site MEL and all introduced sites. Owing to the heuristic nature of ADMIXTURE, we also plotted *K* = 3–5, which recovered further potential structure within the introduced range, separating South African sites and the Eastern Pacific from those in the Atlantic and Mediterranean and blurring the initially inferred structure (electronic supplementary material, figure S3A). The PCA identified four main clusters, each corresponding to one of the four Australian sites (AL, BU, MEL and MAN) with individuals from all introduced sites clustered with those individuals from Melbourne. The first axis of the PCA recovered groupings matching the ADMIXTURE result at *K* = 2 (electronic supplementary material, figure S4A). This close relationship between MEL and the introduced sites was reinforced by the pairwise genetic differentiation *F*_ST_ values (electronic supplementary material, figure S5A).

A greater separation of clusters was identified in *C. robusta* than *M. squamiger*, as seen in the both the higher optimal value of *K* in the ADMIXTURE analysis (figure 3*b*), and PCA (electronic supplementary material, figure S4B)*.* The ADMIXTURE analysis recovered three distinct genomic clusters, with one group containing the native range and NEL, a second group containing the European sites (RAV and PLY) and the western South Africa sites (SB, TB and HB), with the third group containing the eastern South Africa sites (KNY, PE and EL) (figure 3*b*). Interestingly Australian site MEL contained individuals composed of all three clusters (figure 3*b*). Unlike *M. squamiger*, increasing values of *K* did not result in blurring of inferred structure (electronic supplementary material, figure S3B). The PCA recovered a similar picture, however it recovered four clusters (electronic supplementary material, figure S4B). Individuals collected from the northwest Pacific (i.e. the native range) once again clustered together, individuals from South Africa were found in two clusters, corresponding to either the east (KNY, PE and EL) or west coast (SB, TB and HB), and both the site within the English Channel (PLY) and the site within the Mediterranean Sea (RAV) clustered closely to the western South African cluster. However, the PCA recovered the site from New Zealand as a unique cluster (NEL), and genotypes from the Australian site (MEL) encompassed all clusters except the native range (electronic supplementary material, figure S4B). Considering population differentiation (see electronic supplementary material, figure S5B), northwest Pacific sites were genetically similar (average *F*_ST_ = 0.01), but strongly differentiated from other sites (average *F*_ST_ = 0.13).

Regarding the correlation between historical shipping and genomic differentiation, values of *F*_ST_ were slightly negatively correlated with average shipping intensity between 1750 and 2000, though not significantly (electronic supplementary material, figure S6), for both species.

### (e) Inference of colonization routes

Preliminary analyses showed that 2000 simulated datasets per model were suitable for inferring model choice by computing error metrics from both the entire training set and a subset. Likewise, evaluations for each DIYABC-RF run showed that the number of RF trees produced for each model set was sufficient (i.e. error rates stabilized with increasing number of trees). We thus tested a comprehensive variety of models for each species (electronic supplementary material, figures S7 and S8).

**Figure 4.**
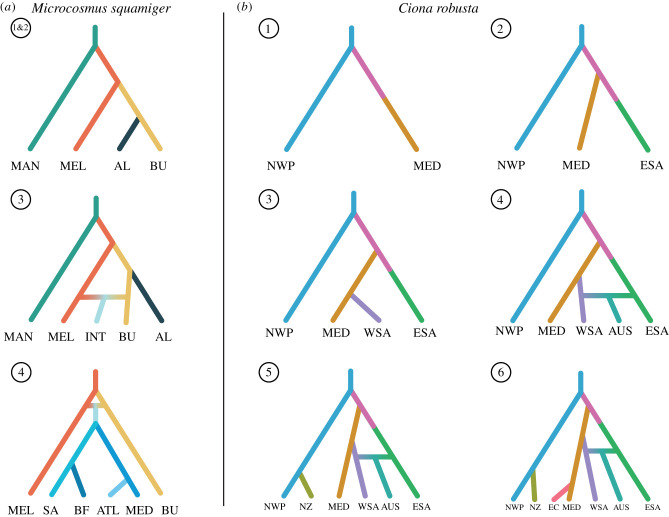
Models of invasion routes identified as most likely using Approximate Bayesian Computation implemented in DIYABC-RF v. 1.0 for the study species (*Microcosmus squamiger* and *Ciona robusta*). Progression through tree is backwards in time, so labelled terminal branches are present day. Numbers in circles indicate scenario set, as in Methods (but see electronic supplementary material, figures S7 and S8 for visual representation of all models), with each set increasing in complexity. Labels are as follows: (*a*) MAN, Manly; MEL, Melbourne; AL, Albany; BU, Bunbury; INT, all introduced sites; SA, grouped sites from South Africa (Mossel Bay, Knysna, Port Elizabeth, Port Alfred, East London and Richards Bay); MED, grouped sites from the Mediterranean (Cubelles, Port Barcelona, Mataró and Arenys de Mar); ATL, grouped sites from the Atlantic (Azores, Santander, Cascais, Cádiz and Chiclana); BF, Bahía Falsa; (*b*) NWP, grouped sites from northwest Pacific (Fukuoka, Busan, Pohang, Tongyeong); MED, Ravenna; ESA, grouped sites from eastern South Africa (Knysna, Port Elizabeth, East London); WSA, grouped sites from western South Africa (Saldanha Bay, Table Bay, Hout Bay); AUS, Melbourne; NZ, Nelson; EC, Plymouth. (Online version in colour.)

For *M. squamiger,* DIYABC-RF was able to confidently identify a split between western and eastern Australian sites (electronic supplementary material, figure S7.1 and table S7, models 17 and 18), followed by admixture between the western site AL and an eastern site MEL. This admixture originated the other western site BU (figure 4*a* model 2; mean posterior probability = 0.601—note the mean prior and mean posterior error rates for the chosen model were high (0.476 and 0.400 respectively, electronic supplementary material, table S8)). Strong evidence of admixture between MEL and BU (figure 4*a* model 3) was also found. Though the final colonization to the introduced range was unresolved, a consensus of potentially suitable models included a split between SA and MED (see the mean number of votes and standard deviations per model in electronic supplementary material, table S7, and posterior probabilities and error rates in electronic supplementary material, table S8), with these two populations being a bridgehead for the BF and ATL populations, respectively.

Regarding *C. robusta,* the DIYABC-RF found that NWP split initially from an unsampled population, with MED (figure 4*b* 1) and ESA (figure 4*b* 2) also being sourced from unsampled populations. WSA was found to be sourced from the MED group (figure 4*b* 3), AUS was identified to be a result of admixture between the east and west coasts of South Africa (figure 4*b* 4), and NZ was recovered to be a secondary introduction from NWP (figure 4*b* 5). The most recent grouping, EC, was identified as sourced from MED (figure 4*b*). See electronic supplementary material, table S9 for posterior probabilities and error rates, and electronic supplementary material, table S10 for the mean number of votes and standard deviations per model.

## 4. Discussion

Our results provide evidence that putatively varying levels of anthropogenic transport do not preclude our ability to recover patterns of population structure across species ranges that have undergone complex introduction histories. Such patterns would not be expected if anthropogenic transport consistently eroded the geographical distribution of genotypes across the species' ranges, and effectively homogenized genomic divergence across the species’ ranges. Additionally, we showed that differing histories of anthropogenic transport can provide a suitable explanation for observed genomic differences between native and introduced ranges.

### (a) Historical patterns of shipping intensity and connectivity

Our temporal analysis of historical shipping networks showed a clear pattern of increasing complexity and intensity of shipping with time [61]. In addition, the results supported our initial assumption that the two studied species have been affected by different levels of anthropogenic transport. Both shipping data and species records suggested that *C. robusta* was subject to anthropogenic transport earlier than *M. squamiger*, providing more opportunities to be redistributed from its original range and a greater time to differentiate from the source populations. For example, the putative native range of *C. robusta* (the northwest Pacific), was an important region for shipping throughout all the time periods studied, becoming a sizeable contributor to shipping from the mid-nineteenth century (figure 2). The observed patterns of historical shipping suggest that *C. robusta* was initially transported during a time with lower shipping intensity and connectivity among distant regions. Regarding the native range of *M. squamiger* (i.e. Australia), it appeared in our initial time period (1750–1800) but was not present again until 1854–1900 (figure 2), suggesting that in the early nineteenth century Australasia may not have been an important source or recipient of global shipping from or to the other study regions. By the time *M. squamiger* was being transported, shipping patterns were complex and thus one source population could spread quickly throughout the introduced range, possibly through a stepping-stone dispersal, which could explain the inferred high levels of population connectivity across much of the introduced range of *M. squamiger*. This may have subsequently led to an increased likelihood of repeated introductions [22]. Such a situation could have occurred when the Suez Canal opened in 1869, rapidly reducing the importance of South Africa as a transportation hub, as seen in the reduction of shipping intensity in the region between 1851 and 1900 (figure 2*c*). Footprints of founder effects, such as the reduction in genetic diversity observed in some *C. robusta* populations, could be explained by introductions of few individuals into the introduced range (as in [62]).

A fundamental assumption made in interpreting our results was the close association between NIS’ introductions and shipping intensity. It would be unreasonable to assume every ship included in our dataset of shipping intensity would lead to an introduction, and our data cannot resolve the magnitude of ongoing, recurrent introductions. However, a higher intensity of ship traffic increases the likelihood of individuals being transported along a certain route and makes it therefore more likely that individuals colonize new sites [61,63]. Indeed, ascidians have been identified in approximately 6% of the ballast waters of ships sailing from the western Pacific to eastern Pacific coastlines [64], and over time such a percentage will likely lead to high levels of propagule pressure. Our analyses including shipping dynamics were limited by the availability of historical data. Shipping data were obtained from two independent datasets spanning two different time periods (i.e. before and after 1850), which differ in their spatial coverage and comprehensiveness. While the early dataset (CLIWIC) has a stronger focus on the Atlantic region, the latter (ICOADS) provides a more comprehensive global coverage, which explains the abrupt changes of shipping dynamics among time periods. Despite these caveats, the datasets gave a good representation of the overall development of the shipping network [65].

### (b) Genomic patterns within native ranges

Species' native ranges are expected to show a clear population structure [66] as the accumulation of mutations [67], genetic drift [68] and/or development of geographical barriers [69] increase population differentiation and the frequency of private alleles over time [70]. Our analyses recovered separate genomic clusters within the native range of *M. squamiger*, with the number of clusters ranging between two and three depending on the analysis. Additionally, the number of private alleles present within sites within the native range was approximately six times greater than those found in the introduced range (electronic supplementary material, table S5). By contrast, the putative native range of *C. robusta* showed limited population structure. This could be due to high levels of gene flow within the native range [71], high effective population size [72] or insufficient sampling. Indeed, it is known that *C. robusta* can be found further east along the coast of Japan than the sampling conducted here [42]. Despite this, the sites from the NWP in the present study portray a similar picture to that from Bouchemousse *et al*. [42], that is, the NWP sites are similar to each other, though are reasonably genomically diverse too. Further sampling across the NWP would provide clarification as to whether the genomic homogeneity present in the native range is due to the genomic homogenization of populations within the native range through anthropogenic transport [73].

### (c) Genomic patterns within introduced ranges

While genetic bottlenecks are often mentioned in the literature of biological invasions [74], it is becoming increasingly appreciated that introduced populations do not regularly undergo a significant reduction in genomic diversity [20]. Multiple introductions [75], high gene flow [76] and/or genetic admixture [77] often overcome any reduction in genetic diversity associated with bottlenecks. We did not find evidence of a reduction in genomic diversity between the native and introduced range of *M. squamiger*, possibly either due to increased propagule pressure owing to intense anthropogenic transport, or genetic admixture between native sites (see results of the DIYABC analyses). The extensive introduced range of *M. squamiger* was highly homogeneous, both in terms of population structure and genomic diversity patterns. Global genomic homogeneity within the introduced range could be the result of the introduction of genotypes from a single-source population from the native range [78] or high levels of population connectivity within the introduced range due to intense anthropogenic transport [79] promoting stepping-stone dispersal. By contrast, we found evidence of population structure within the introduced range of *C. robusta*. Population structure within introduced ranges has been found in other ascidians [80], and can be attributed to multiple independent introduction events [62]. The observed population structure in *C. robusta* was present at differing spatial scales. For example, geographically distant regions such as Europe and western South Africa were genomically homogeneous, supporting previous results found by Zhan *et al*. [79]. Historical records of *C. robusta* identify the ascidian as being present along the western coast of South Africa since the 1950s [81]. Whether the observed similarity in genomic makeup between these two regions is a result of ongoing anthropogenic transport, or the result of high *N*_e_ supressing the effects of genetic drift, remains unknown, though we recovered a drop in H_E_ in western South Africa sites compared to those found in eastern South Africa (electronic supplementary material, figure S2). It is unclear whether the limited natural dispersal potential of ascidians, coupled with their affinity to inhabit artificial environments (i.e. marinas, ports, harbours), has an effect on *N*_e_. However previous work on the congener *C. savignyi* showed a large effective population size as inferred in San Francisco Bay [82]. On a regional scale, we found clear structure along the South African coastline. Strong regional differentiation in South Africa could be due to demographic processes or introductions from multiple independent source populations. Regarding genomic diversity, we observed a decrease from the putative native range to western South Africa populations, which may provide evidence for demographic processes contributing to genomic differentiation. As *C. robusta* has been present along the western coast of South Africa since at least the 1950s [81], it is unlikely that the low levels of genomic diversity is the result of a recent introduction. Finally, the DIYABC-RF analyses identified different introduction sources for both the eastern and western coasts of South Africa. Taken together, *C. robusta* displays population structure in South Africa likely due to existing marine biogeographic provinces, demographic processes and/or independent introductions.

### (d) Reconstructing invasion routes

The species with the shorter history of anthropogenic transport, *M. squamiger,* showed limited confidence in the reconstruction of invasion routes, with only one scenario set having a prior error rate of less than 45% (electronic supplementary material, table S8). In accordance with previous work using microsatellite and DNA sequence data [31], we found strong evidence that *M. squamiger* is native to Australia. Furthermore, we found evidence that the genomic homogeneity of the introduced range of *M. squamiger* resulted from a single-source introduction from an unsampled site comprising individuals from either Melbourne or from admixture between Melbourne and Bunbury sites, with subsequent stepping-stone dispersal. Such a signature of high homogeneity across the introduced range has been observed in other marine organisms. For example, genetic homogeneity has been identified within the introduced range of the invasive lionfish (*Pterois volitans*) with the conclusion that gene flow can quickly erode previous signals of genetic divergence [13].

While we found evidence of population structure between introduced populations of *M. squamiger* and the native range outside Melbourne, we could not discount the possibility of introduced alleles re-entering the native range. This is suggested by the discord between the clustering and the DIYABC-RF analyses, with the former indicating that Melbourne was the sole source. Further evidence for Melbourne being the source of all the introduced populations came from the fact that the lowest number of private alleles across all native sites were found in Melbourne. We know from historical data that Melbourne and Bunbury opened as ports from the 1850s onwards [83,84], and just over a century later *M. squamiger* individuals were found in California [85] and the Mediterranean Sea [39]. This was reinforced by our shipping history data, which showed that Australia only started increasing its shipping activity from the 1850s, and indeed only became a significant global contributor after the 1900s. This further indicates that over the twentieth century, *M. squamiger* colonized distant regions around the globe, demonstrating how rapidly anthropogenic transport can facilitate the establishment and spread of NIS.

Poorly documented species records from the literature posed a challenge for guiding our analyses of the colonization history of *C. robusta*. While the prior error rates of the scenario sets were lower (i.e. higher confidence in model choice) than those for *M. squamiger*, they still ranged between 14 and 36% (electronic supplementary material, table S9). This in part may be the reason why our DIYABC-RF analyses were unable to identify the source of the Mediterranean and eastern South Africa sites (both coming from an unsampled population). In turn, we were able to find evidence for multiple introductions and potential admixture (e.g. figure 4) events promoting the expansion of the species. A previous genetic study of *C. robusta* also sampled a large part of the species range [42] and found that, in line with previous work, the northwest Pacific is the putative native range; although an introduced status of *C. robusta* in the northwest Pacific could not be disproved based on their evidence, consistent with the results presented here.

Until recently, little has been known regarding the effects of anthropogenic transport on genetic patterns across species ranges, but a growing number of studies are unravelling invasion routes despite an intensification of anthropogenic transport in recent decades/centuries (figure 2). For example, Manni *et al.* [14] were able to accurately define the source populations of the Japanese Asian tiger mosquito (*Aedes albopictus*) despite exhibiting chaotic propagule dispersion associated with trans-continental anthropogenic transport. Similarly, Lesieur *et al.* [86] found that despite a complex invasion history and long-distance dispersal owing to anthropogenic transport of species, the invasion pathway of the Western conifer seed bug (*Leptoglossus occidentalis*) could still be tracked. Our genomic results showed that invasion routes of NIS with high historical anthropogenic transport can be studied with similar confidence as NIS with both shorter residence times in the introduced range, and lower levels of anthropogenic transport. We therefore conclude that although considering anthropogenic transport remains important, it does not preclude inference with genomic data, providing that sampling is of sufficient geographical breadth.

With anthropogenic transport of species being a major factor dictating the distribution of many range-shifting species [4,7,87], it is essential to consider artificial connectivity pathways among populations to plan both management and mitigation actions [88]. Specifically, knowledge of source/s of prolific range-shifting populations may aid planning management actions such as vector/NIS eradication. Our study results unravelled how anthropogenic transport changes the geographical distribution of genetic lineages, as well as provided applied knowledge particularly relevant to stakeholders with an interest in mitigating the effects of NIS.

## Data accessibility

The data for this study have been deposited in the European Nucleotide Archive (ENA) at EMBL-EBI under accession no. PRJEB46018 https://www.ebi.ac.uk/ena/browser/view/PRJEB46018.
